# Supramodal executive control of attention

**DOI:** 10.3389/fpsyg.2015.00065

**Published:** 2015-02-24

**Authors:** Alfredo Spagna, Melissa-Ann Mackie, Jin Fan

**Affiliations:** ^1^Department of Psychology, Queens College, City University of New York, New York, NYUSA; ^2^The Graduate Center, City University of New York, New York, NYUSA; ^3^Department of Psychiatry, Icahn School of Medicine at Mount Sinai, New York, NYUSA; ^4^Department of Neuroscience, Icahn School of Medicine at Mount Sinai, New York, NYUSA

**Keywords:** attentional networks, visual attention, auditory attention, alerting, orienting, executive control

## Abstract

The human attentional system can be subdivided into three functional networks of alerting, orienting, and executive control. Although these networks have been extensively studied in the visuospatial modality, whether the same mechanisms are deployed across different sensory modalities remains unclear. In this study we used the attention network test for the visuospatial modality, in addition to two auditory variants with spatial and frequency manipulations to examine cross-modal correlations between network functions. Results showed that among the visual and auditory tasks, the effects of executive control, but not effects of alerting and orienting, were significantly correlated. These findings suggest that while alerting and orienting functions rely more upon modality-specific processes, the executive control of attention coordinates complex behavior via supramodal mechanisms.

## INTRODUCTION

The human brain receives enormous amounts of sensory stimuli from visual, auditory, and other sensory modalities. Thus, allocation of mental resources to efficiently process input information, implemented via attentional mechanisms, is critical (Mackie et al., 2013). In human social interactions, we are able to selectively attend to the voice of one person, while filtering irrelevant sound sources from the environment. This is a phenomenon known as the “cocktail party effect” (Cherry, 1953). Modern investigations of selective attention began with experiments on audition (Broadbent, 1958) with findings demonstrating that neurons in auditory cortex can be strongly modulated by attention (Hubel et al., 1959). However, much existing attention research has been focused on the visual modality (Posner and Petersen, 1990; Klein, 2000; Fries et al., 2001), due in part to its status as the “dominant” modality, exerting great influence over signals in other modalities (Posner, 1978). Auditory attention allows for the selection of auditory streams of interest (Fritz et al., 2007). This selection process is similar to the manner in which visual stimuli of interest are selected in the environment (Shinn-Cunningham, 2008). Although considerable effort has been devoted to investigate whether the same attentional mechanisms are involved irrespective of modality of the stimuli (Spence et al., 2000; Spence, 2010), the existence of “supramodal” attentional functions remains unclear. This is primarily due to a lack of studies employing comparable visual and auditory attention tasks to examine the relationship of attentional effects across modalities (Spence and Driver, 1994; Zatorre et al., 1999; Salmi et al., 2007; Larson and Lee, 2014).

One view of attention refers to the activity of a set of brain networks composed of alerting, orienting, and executive control which influences the priority of domain-specific information processing (Fan et al., 2009). These attentional networks are responsible for producing and maintaining a state of readiness in order to process non-specific impending inputs (alerting function), selecting the most relevant information from various inputs within and across modalities (orienting function), and detecting and resolving conflict among competing mental processes (executive control function) to make rapid and accurate responses (Posner and Petersen, 1990; Fan et al., 2002, 2005). Within this framework, the cocktail party effect can be decomposed into (1) phasic alerting that occurs when one hears, for example, a familiar name in conversation; (2) orienting to the location and speaker of interest, in order to attend to the message presented (Broadbent, 1958); and (3) executive control of attention to inhibit competing messages from other sources. In this way, the attention functions allow for more efficient processing of myriad auditory inputs from various streams (Fritz et al., 2007).

In visual attention studies, the alerting function is commonly elicited by presentation of a non-specific warning cue preceding the target (Fan et al., 2002, 2009). The warning cue leads to a faster reaction time (RT) compared with when no cue precedes the target (Petersen and Posner, 2012). Most of the studies investigating both visual and auditory alerting showed benefits with visual and auditory warning cues, though auditory targets generally benefited less than visual targets due to automatic alerting effects of sounds (Fernandez-Duque and Posner, 1997). Evidence for the existence of modality-specific mechanisms of the alerting function comes from a study investigating the relationship between visual and auditory cueing, which showed that these effects were not correlated (Posner, 1978; Roberts et al., 2006). Although many aspects of the paradigms used may have been responsible for the lack of correlation between the auditory and visual alerting effects, this result might be better explained by the nature of alerting cues which are first encoded in primary sensory cortex, and therefore are tied to modality-specific mechanisms (De Santis et al., 2007).

The orienting of attention has been mostly studied in the visuospatial (VS) domain. These studies used variants of the Posner spatial cueing paradigm (Posner, 1980), where a cue correctly (valid cue) or incorrectly (invalid cue) predicted the target location. There is extensive literature addressing the benefit and cost in both RT and accuracy for valid versus invalid cue trials (the validity effect; see Macaluso and Doricchi, 2013 for a review). Paralleling the visual modality, auditory attention can be directed to a variety of stimulus features, e.g., spatial location, frequency, speech *vs.* non-speech streams (Fritz et al., 2007). While early attempts to study auditory spatial (AS) attention did not reveal a beneficial effect for targets following spatial cues (Posner, 1978; Buchtel and Butter, 1988), validity effects in the auditory modality have been found for both spatial (Spence and Driver, 1994; Mayer et al., 2009; Santangelo et al., 2009) and non-spatial cues (Posner, 1978; Mondor and Bregman, 1994). The majority of comparisons between attention in visual and auditory modalities have been focused on the orienting function, with some evidence emphasizing the presence of a shared frontoparietal network for both modalities (Shomstein and Yantis, 2006; Wu et al., 2007). However, these experiments measured neural events related to voluntary orienting using tasks confounded by the involvement of executive control of attention, thus making it difficult to draw conclusions about the modality-dependent or independent nature of orienting. Conversely, a study examining the relationship between visual and auditory exogenous orienting functions showed that these effects were not correlated (Roberts et al., 2006). However, in this experiment, spatial orienting benefits were obtained only in the visual task, but not in the auditory task.

The executive control of attention is a mechanism that is responsible for detecting and resolving conflicts among mental processes (Fan et al., 2002). It is usually examined using tasks involving conflict among stimulus and response dimensions, such as the flanker task (Eriksen and Eriksen, 1974) and the Simon task (Simon and Berbaum, 1990). While the terms executive control and executive function are often used interchangeably in the literature, for the purpose of this study we refer exclusively to the executive control of attention, which underlies, but is not equivalent to, the broader concept of executive functions (Mackie et al., 2013). There exists a wealth of studies which investigate the mechanisms of executive control in different modalities (see Roberts and Hall, 2008 for a review). Functional neuroimaging studies showed the activity of the frontoparietal network in response to both visual (Fan et al., 2003, 2005; Blasi et al., 2006) and auditory conflict tasks (Roberts and Hall, 2008; Donohue et al., 2012). However, evidence for significantly correlated effects of executive control across modalities have only been found for the Stroop task (Roberts et al., 2006), and not for other tasks. The ability to inhibit task-inappropriate responses and unwanted information is central to executive control, but whether the same executive control mechanisms are deployed independent of modality needs to be further investigated.

The three attentional functions may have a hierarchical structure, with executive control at the highest level (possibly supramodal) and alerting and orienting at a lower level (possibly modality-specific; Wang and Fan, 2007; Mackie et al., 2013). The benefit of this hierarchical structure may be understood within the context of the cocktail party effect. Alerting and orienting rely on lower levels of processing, such that the encoding of sensory information in primary sensory cortices drives modality-specific attention. On the other hand, executive control of attention, at a higher level of attentional processing, coordinates mental computations and integrates information across modalities. Modality-specific executive control of attention would be an inefficient use of mental resources, as a further higher-level mechanism would still be required to integrate across modalities. Evidence for the existence of supramodal or modality-specific mechanisms of the attentional networks is still elusive. Previous investigations have studied each attentional function in isolation, yet methodological differences between tasks purportedly eliciting the same function in different modalities have made cross-study comparisons difficult.

To characterize the supramodal and modality-specific mechanisms of the attentional functions, we need to differentiate between sources and sites of attentional control (Posner and Driver, 1992). The sources of the attentional mechanisms are defined as the cortical areas that are responsible for producing the effect, while the sites are the areas where the attentional modulation is implemented. For example, while the sites of auditory or visual stimulation are the respective primary sensory cortices receiving the input from the environment, the source of the attentional modulation may be the same (i.e., supramodal). Therefore, attentional effects may rely on a combination of supramodal sources and modality-specific sites (Corbetta, 1998; Driver and Frackowiak, 2001; Miller and Cohen, 2001). If the attentional effects are highly correlated across two modalities, this correlation must be driven by the existence of supramodal mechanisms, because the sites are necessarily modality-specific.

The aim of the present study is to investigate whether the attentional functions are supramodal or modality-specific by examining correlations between the attentional network effects in the auditory and visual modalities. We hypothesized that executive control shares attentional resources across the visual and auditory modalities, while alerting and orienting require modality-specific resources. To test this hypothesis, we used the Attention Network Test (ANT; Fan et al., 2002) and two auditory variants with spatial and frequency manipulations to examine within-subject correlations between attentional functions across tasks. The ANT has been widely used to measure the efficiency of the alerting, orienting, and executive control of attention in the visual modality. The revised version of this task, the ANT-R (Fan et al., 2009), magnifies the interactions among the attentional functions by additionally manipulating the validity of spatial cues and the cue-to-target intervals. We designed an auditory version of the ANT-R where AS cues were used to parallel the visual task. Due to the frequency-based organization of the auditory cortex, auditory attention can also be directed toward frequency properties of stimuli. Therefore, we developed a second auditory version where the frequency of the cue may indicate the exact frequency of an upcoming target. The three tasks used in this study were intended to elicit the alerting, orienting, and executive control functions, as well as their interactions, in the VS, AS, and auditory frequency (AF) modalities or domains. If an attentional effect is positively correlated across modalities, it would support the existence of a supramodal mechanism for that specific attentional function. In line with previous evidence, we expected that the executive control effects, rather than alerting and orienting effects, would be significantly correlated across tasks.

## MATERIALS AND METHODS

### PARTICIPANTS

Forty-two participants took part in this study (27 females; mean age 22.1 ± 3.8 years; range 18–29 years), and were compensated for a 2-h session. Written informed consent approved by the Institutional Review Board of the City University of New York was obtained from each participant.

### THE VISUOSPATIAL ATTENTION NETWORK TEST

**FIGURE 1:**
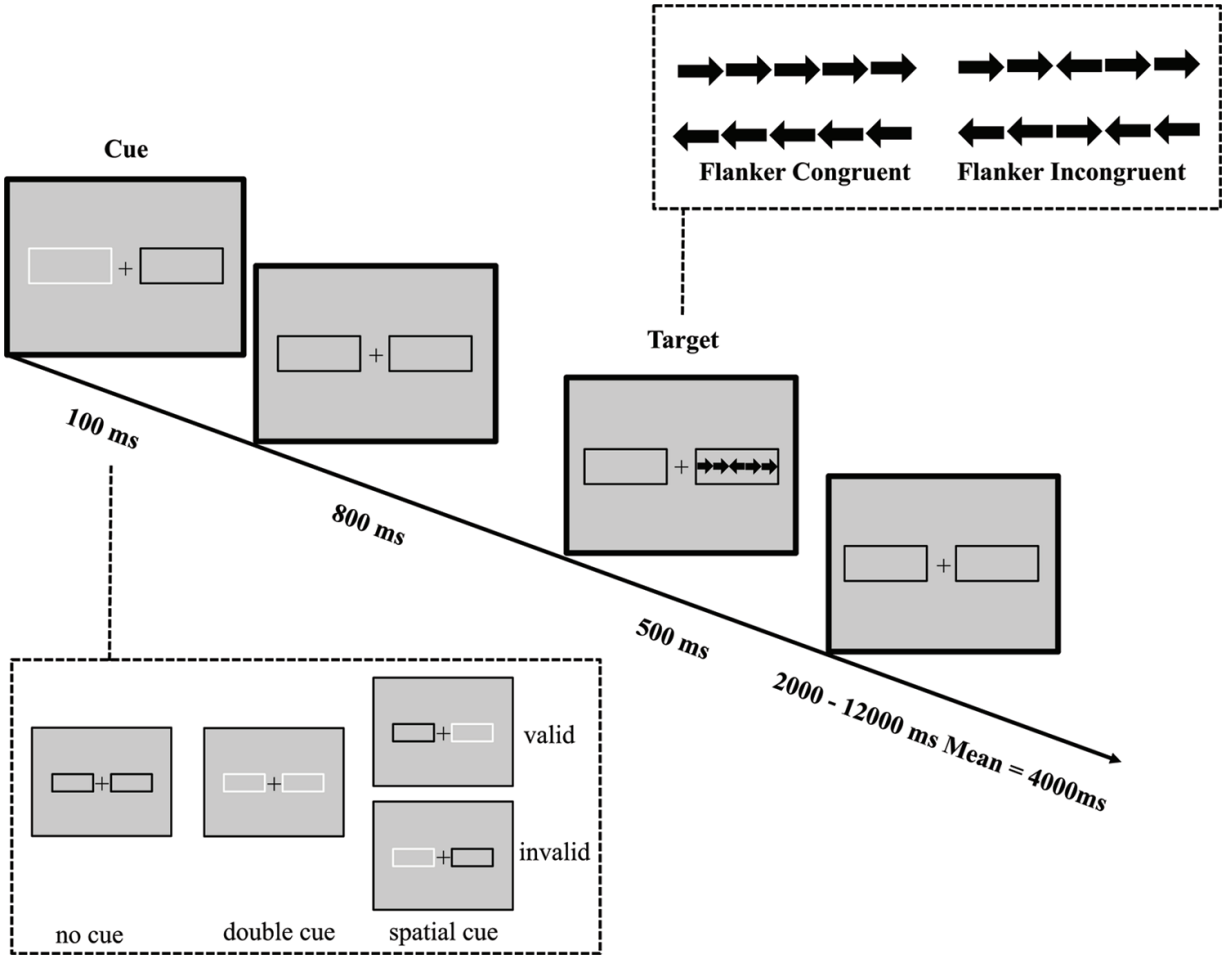
**Schematic of the visuospatial ANT (ANT-VS).** In this task participants made responses to indicate the direction of a central arrow (left or right) which was surrounded by two flanker arrows on each side pointing either to the same direction as the target (congruent condition) or to the opposite direction (incongruent condition). Before the target appeared, a cue in the form of a box flashing on one or both sides was displayed. The cue could be valid, which predicted the target position correctly, or invalid, which predicted the opposite position. Also, there was a double cue condition, in which both boxes flashed, to provide temporal but not spatial information, and in the no cue condition no cue was presented.

We used a revised version of the ANT-R, with a fixed 800 ms cue-to-target interval (see **Figure 1** for an illustration of the visuospatial ANT, ANT-VS). A long SOA was used to allow enough time for the auditory cueing effect to occur and to avoid overlap between the cue and the target. Therefore, to use the same cue-to-target interval in the three tasks, we modified the original ANT-R version by keeping the SOA constant at 800 ms, which generated a reliable cueing effect in our previous study (Fan et al., 2009). A fixation cross was displayed at the center of the screen throughout the experiment. On every trial, participants saw a row of five black arrows presented for 500 ms against a gray background. The central arrow was the target, and the other four arrows were flankers. The target arrow pointed to the left or right, and the flanker arrows pointed either in the same direction as the target arrow (congruent condition) or in the opposite direction (incongruent condition), with equal probability. Participants were asked to identify the direction of the target arrow with a button press and to respond as quickly and as accurately as possible. The response time window was 1700 ms after the onset of the target. The duration between the offset of the target and the onset of the next trial was varied systematically, approximating an exponential distribution ranging from 2000 to 12,000 ms and having a mean of 4000 ms (10 intervals from 2000 to 4250 ms with an increase step of 250 ms, then one 4750 ms interval and one 12,000 ms interval). The mean trial duration was 5000 ms.

The arrows appeared at one of two locations to the left and right of a central fixation cross. The presentation of the arrows was preceded by a visual cue on most trials. Thin black frames surrounded these locations, and one or both of these frames were briefly flashed as the cue by changing the frame from black to white for 100 ms. There were three different types of cues: (1) no cue (no flash prior to target onset); (2) double cue (flashed in both locations); and (3) spatial cue (one location flashed prior to target onset). The characteristic distinguishing between the double cue condition and the no cue condition is that in the former, the cue provides information about when the target is going to appear, while in the latter condition no cue is presented. The difference between these two conditions gives a measure of how temporal information regarding the upcoming target benefits participants’ performance. The spatial cue provided both temporal and spatial information about the target arrow, with 75% of the spatial cues in the same location as the upcoming targets (valid cue), and the remaining 25% of the spatial cues in the opposite location (invalid cue). The difference between these two conditions gives a measure of how valid spatial information about the upcoming target benefits participants’ performance, compared to a performance cost by invalid spatial information. Participants completed four runs, each with 72 trials, for a total of 288 trials. Of the total trial number, there were 48 trials for the no cue condition, 48 trials for the double cue condition, 48 trials for the invalid cue condition and 144 for the valid cue condition. Each run lasted approximately 750 s.

The alerting function is measured by the difference between the no cue and double cue conditions. The orienting function is measured by the difference between double cue and valid cue conditions. The validity effect of orienting is measured by the difference between valid and invalid cue conditions. The executive control function is measured by the difference between incongruent and congruent flanker conditions, defined as the conflict effect. See **Table 1** for the operational definitions of the attentional effects and interactions.

**Table 1:** Operational definition of the attentional network effects and interactions as differences between conditions.

	Testing condition	*Minus*	Reference condition
**Network effects**
Alerting	No cue		Double cue
Orienting	Double cue		Valid cue
Validity	Invalid cue		Valid cue
Conflict	Incongruent		Congruent
**Interactions**
Alerting by Conflict	No cue, incongruent *minus* No cue, congruent		Double cue, incongruent *minus* double cue, congruent
Orienting by Conflict	Double cue, incongruent *minus* Double cue, congruent		Valid cue, incongruent *minus* Valid cue, congruent
Validity by Conflict	Invalid cue, incongruent *minus* Invalid cue, congruent		Valid cue, incongruent *minus* Valid cue, congruent

### THE AUDITORY SPATIAL CUE ATTENTION NETWORK TEST

**FIGURE 2:**
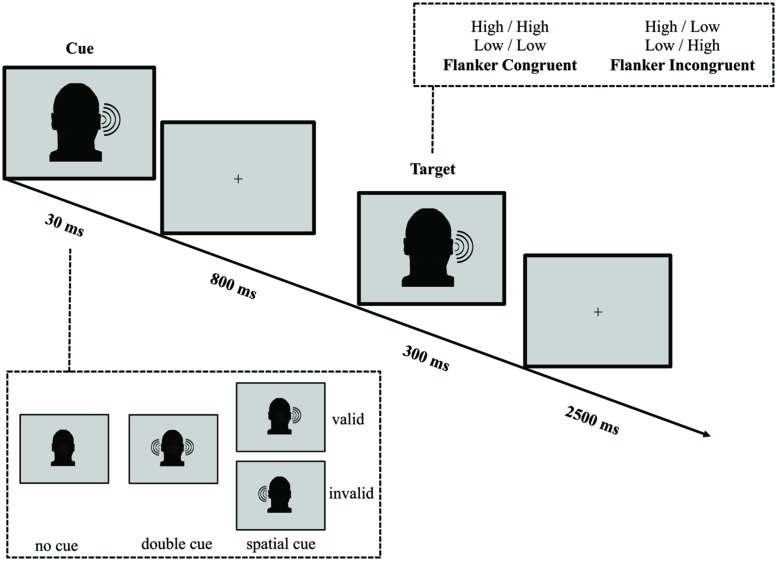
**Schematic of the auditory spatial ANT (ANT-AS).** In this task participants made responses to indicate the pitch (high or low) of a target tone presented to the left or the right ear, which was followed by the presentation of another tone either of the same frequency (congruent condition) or of a different frequency (incongruent condition). Before the target appeared, a cue in the form a single tone presented on one or both ears, might indicate the position where the target, a high or low tone, would subsequently appear. The cue could be valid, which predicted the target position correctly, or invalid, which predicted the opposite position. Also, there was a double cue condition, in which a tone was presented in both ears, and the no cue condition, where no cue was presented.

In parallel with the ANT-VS, we designed the ANT-AS to evaluate the auditory attentional network functions when AS cues are provided (see **Figure 2** for an illustration of this task). Auditory stimuli were created using Audacity (GNU/GPL License; Sourceforge.net) at a sampling rate of 44.1 kHz, and presented at 80 dB SPL. On every trial, participants listened to two 100 ms tones monaurally presented. The tones, presented to the same ear (left or right), were separated by 100 ms of silence, and were either high (1250 Hz) or low (750 Hz) in frequency. The first tone was defined as the target, and the second tone was defined as an irrelevant flanker. Congruence and incongruence between the target and flanker tones were achieved by having the two tones the same (high–high, low–low) or differ (high–low, low–high) in frequency. Participants were instructed to report the category of the tone in frequency (high or low) of the target, with a button press, within a 2500 ms response window after the onset of the target. A fixation cross was displayed at the center of the screen throughout the experiment.

A 30 ms click sound cue was variably presented 800 ms before the onset of the target tone. The cue conditions and trial numbers were analogous to those in the ANT-VS: (1) no cue (no click prior to the target); (2) double cue (click presented to both ears); and (3) spatial cue (click presented to only one ear). The cue validity was also manipulated and the attentional effects were defined as those in the ANT-VS.

The average duration between target offset and onset of the next trial was 1800 ms (four intervals: 1200, 1600, 2000, and 2400 ms). The mean trial duration was 4000 ms. In each experimental run, participants responded to 72 test trials each over four runs, with a mean run duration of 420 s.

### THE AUDITORY FREQUENCY-CUE ATTENTION NETWORK TEST

**FIGURE 3:**
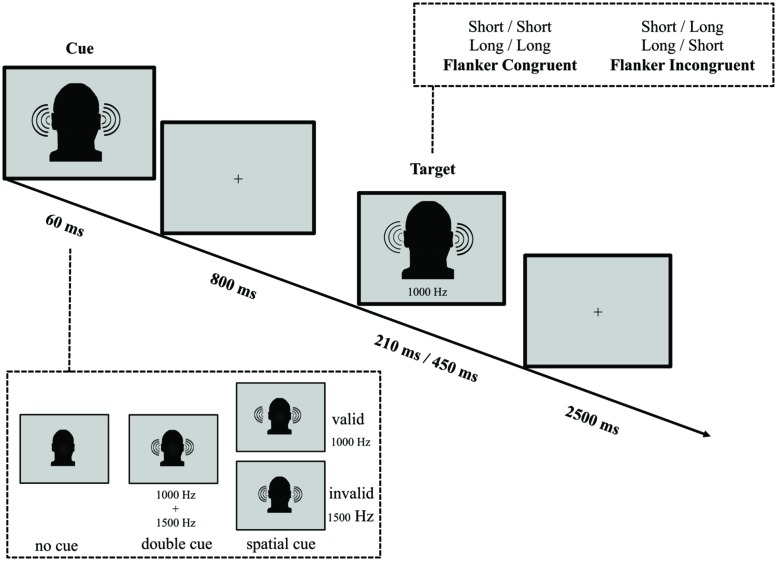
**Schematic of the auditory frequency ANT (ANT-AF).** In this task participants made responses to indicate the duration (short or long) of a target tone, which was followed by the presentation of another tone either of the same duration (congruent condition) or of the other duration (incongruent condition). Before the target appeared, a cue in the form a single tone presented on both ears might indicate the pitch of the upcoming target. The cue could be valid, which predicted the pitch of the target correctly, or invalid, which predicted the other pitch. Also, there was a double cue condition, in which both pitch tones were simultaneously presented, and the no cue condition, where no cue was presented.

In parallel with the ANT-VS and the ANT-AS, we designed the ANT-AF to evaluate efficiency and interactions of the auditory attentional functions when frequency (non-spatial) orienting is involved (see **Figure 3** for an illustration of the task). Because previous work has demonstrated limited benefit from AS cues (Posner, 1978), we were interested in whether frequency-cues would be more effective in eliciting the orienting response, given that the organization of primary auditory cortex is frequency-based. In the ANT-AF, a fixation cross was displayed at the center of the screen throughout the experiment. On every trial, participants listened to two binaurally presented tones in either high (1500 Hz) or low (1000 Hz) frequency, separated by a 150 ms silence interval. Participants were instructed to report if the first of these two tones was short (30 ms) or long (150 ms) in duration by pressing a button within a 2500 ms response window after the onset of the target. The second tone served as a flanker, which could be of the same duration (short–short or long–long) defined as the congruent condition, or of the alternative duration (short–long or long–short) defined as the incongruent condition.

A binaural 60 ms cue tone was variably presented 800 ms before the target. The cue conditions were analogous to those in the ANT-VS: (1) no cue (no tone prior to the target); (2) double cue (a complex tone resulting from the sum of a 1000 plus a 1500 Hz tone); and (3) frequency cue (a 1000 or 1500 Hz pure tone). The frequency of the cue and the frequency of the target could be the same (i.e., cue 1000 Hz and target 1000 Hz or cue 1500 Hz and target 1500 Hz) serving as the valid orienting cue, or the cue and the target were different in frequency (i.e., cue 1000 Hz and target 1500 Hz or cue 1500 Hz and target 1000 Hz) serving as the invalid orienting cue. In valid trials, a cue indicated the frequency of the upcoming target, while in invalid trials the indicated frequency was inconsistent with the target.

The average duration between target offset and onset of the next trial was 1800 ms (four intervals: 1200, 1600, 2000, and 2400 ms), and the mean trial duration was 4500 ms. In each experimental run, participants completed 48 test trials each over six runs, with a mean run duration of 280 s.

The operational definitions of the alerting, orienting, and executive control effects were the same as those in the ANT-VS and ANT-AS. It should be noted that the average inter-trial interval (ITI) and the response window differed between the visual task (4000 and 1700 ms, respectively) and the auditory tasks (1800 and 2500 ms, respectively). Because we used the sequential manipulation of target and flankers, which increases the presentation duration, we opted for a response window that was greater than the one used in the ANT-VS. Further, in order to keep the duration of the three tasks similar (approximately 30 min), we shortened the ITI in the auditory tasks. We compensated for these differences by applying a homogeneous filter on the RT in each task (only RTs between 200 and 1700 ms were included in the analysis) to match the response window of the ANT-VS.

### PROCEDURE

The three tasks were presented on a PC using E-Prime^TM^ 2.0 software (Psychology Software Tools, Pittsburgh, PA, USA). The visual stimuli were presented on a 17-inch LCD screen (Dell, 2007FBp), and the auditory stimuli were presented using headphones (Bose, QuietComfort 15). Five-millisecond fade-in/fade-out ramps were applied to each tone to reduce transient clicks. The order of tasks was counterbalanced across participants. Before each task, a block of 24 practice trials was administered, and at the end of each practice trial visual feedback was displayed for 1 s, reporting response accuracy, and RT.

### DATA ANALYSIS

Mean RT and error rate for each condition were calculated. Error trials (incorrect and missing responses) and RTs below 200 ms and above 1700 ms were excluded from the calculations of mean RT and attentional effects. The attentional effects for RT and error rate were computed using the definitions in **Table 1**. Further details about the rationale behind these formulas can be found in the original paper (Fan et al., 2009). The significance of the effects was tested using two-tailed one-sample *t*-tests. Effect sizes are also reported as Cohen’s *d*. Pearson’s correlation analyses were conducted on the attentional effects across tasks, to account for linear relationships between two attentional effects. Spearman’s correlation analyses were also conducted to examine the degree of monotonic relationship between two effects. The split-half method was used to estimate the reliability of each attentional effect in the three tasks by examining the internal consistency (the correlation) between the first half of trials and the second half of trials.

## RESULTS

**Table 2** shows the RT (±SD) and error rate (±SD) for each experimental condition. The overall RTs for the VS, AS, and AF ANTs were 667 ms (SD = 122), 788 ms (SD = 218 ms), and 786 ms (SD = 176 ms), respectively, while the overall error rates were 7.01% (SD = 5.74%), 6.57% (SD = 5.84%), and 8.17% (SD = 3.20%), respectively. **Table 3** shows the mean, SD, and effect size of the attentional effects in the three tasks, for both RT and error rate.

**Table 2:** Mean reaction time (RT; SD) in milliseconds, and error rate (SD) in percentage, for the visuospatial (VS), auditory spatial (AS), and auditory frequency (AF) tasks.

		Double	Invalid	No cue	Valid
**RT**
VS	Congruent	557 (116)	614 (119)	609 (122)	516 (115)
	Incongruent	735 (150)	830 (148)	815 (153)	657 (144)
AS	Congruent	687 (180)	686 (179)	740 (175)	682 (175)
	Incongruent	859 (281)	586 (281)	929 (255)	866 (279)
AF	Congruent	693 (167)	694 (167)	882 (170)	675 (169)
	Incongruent	779 (211)	767 (201)	1045 (222)	754 (211)
**Error rate**
VS	Congruent	0.69 (2.32)	0.99 (3.28)	1.54 (3.91)	1.36 (2.46)
	Incongruent	12.05 (13.70)	16.72 (15.77)	16.28 (15.03)	9.48 (7.67)
AS	Congruent	2.78 (4.48)	1.98 (3.35)	94.86 (6.63)	2.65 (3.19)
	Incongruent	9.23 (11.10)	9.62 (9.41)	12.20 (14.12)	9.23 (9.33)
AF	Congruent	2.38 (2.63)	3.08 (3.91)	3.27 (5.00)	1.92 (2.06)
	Incongruent	5.95 (5.97)	7.24 (7.25)	36.01 (12.44)	5.25 (5.14)

**Table 3:** Mean, SD, and Effect Size (Cohen’s *d*) for the attentional effects in the three tasks, in both RT and error rate.

	RT (ms)			Error rate (%)		
	Mean	SD	Cohen’s *d*	Mean	SD	Cohen’s *d*
**VS**
Alerting	66	26	2.54	1.91	4.84	0.39
Orienting	60	32	1.88	1.86	4.31	0.43
Validity	136	50	2.72	4.34	5.82	4.82
Conflict	185	80	2.31	11.72	9.82	1.19
A × C	28	51	0.55	2.13	9.05	0.24
O × C	38	44	0.86	5.04	9.48	0.53
V × C	75	42	1.79	9.41	11.38	0.83
**AS**
Alerting	61	63	0.97	2.53	6.45	0.39
Orienting	-1	34	-0.03	0.07	3.94	0.02
Validity	-3	47	-0.06	-0.13	3.88	-0.03
Conflict	179	126	1.42	7.00	8.95	0.78
A × C	16	98	0.16	0.89	12.84	0.07
O × C	-11	72	-0.15	-0.13	7.89	-0.02
V × C	-14	74	-0.19	1.06	7.09	0.15
**AF**
Alerting	228	126	1.81	15.48	6.04	2.56
Orienting	21	38	0.55	0.45	3.35	0.13
Validity	16	41	0.39	1.44	3.10	0.46
Conflict	100	72	1.39	11.02	5.64	1.95
A × C	77	136	0.57	29.17	14.90	1.96
O × C	7	75	0.09	-0.03	6.18	0.00
V × C	-6	68	-0.09	0.56	7.69	0.07

A × C, Alerting by Conflict; O × C, Orienting by Conflict; V × C, Validity by Conflict.

### THE VISUOSPATIAL ATTENTION EFFECTS

**FIGURE 4:**
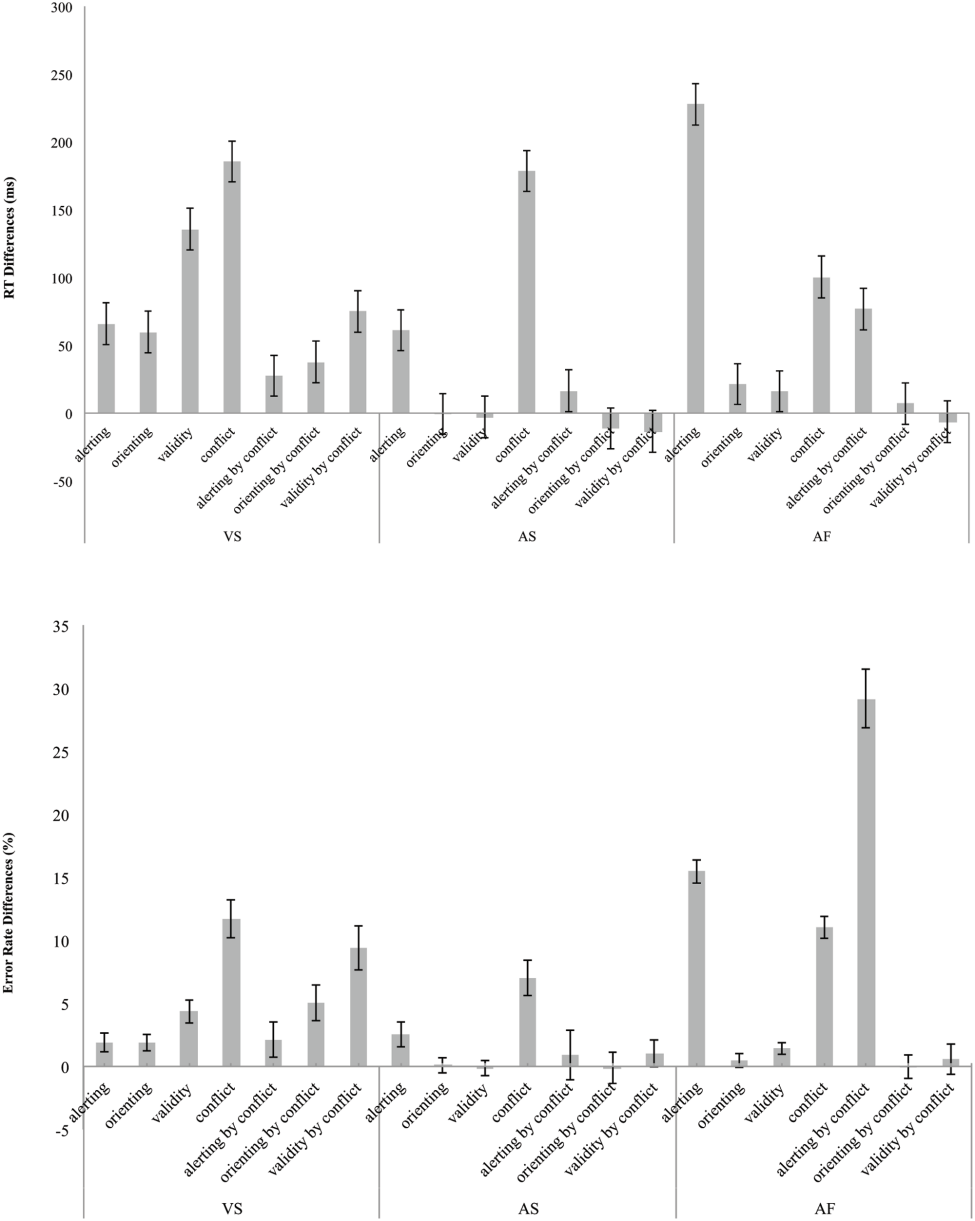
**Attentional network and two-way interaction scores in terms of RT **(top)** in milliseconds and error rate **(bottom)** differences in percentage for the VS, AS, and AF tasks.** The error bars represent SE.

**Figure 4** shows the network effects and interactions in RT (left – top section) and error rate (left – bottom section) for the ANT-VS.

#### The alerting effect

The alerting effect was significant for both RT [66 ± 26 ms, *t*(41) = 16.24, *p* < 0.001] and error rate [1.91 ± 4.84%, *t*(41) = 16.24, *p* < 0.001], indicating that participants were faster and more accurate in the double cue compared to the no cue condition.

#### The orienting effect

The orienting effect was significant for both RT [60 ± 32 ms; *t*(41) = 12.24; *p* < 0.001] and error rate [1.86 ± 4.31%; *t*(41) = 2.80; *p* < 0.01], indicating that participants were faster and more accurate in the valid cue condition compared to the double cue condition.

#### The validity effect

The validity effect was significant for both RT [136 ± 50 ms; *t*(41) = 17.73; *p* < 0.001], and error rate [4.34 ± 5.82%; *t*(41) = 4.84; *p* < 0.001], indicating that the RT was shorter and accuracy was greater for the valid cue condition compared to the invalid cue condition.

#### The conflict effect

The conflict effects in RT (185 ± 80 ms) and error rate (11.72 ± 9.82%) were significant [*t*(41) = 15.03; *p* < 0.001, and *t*(41) = 7.74; *p* < 0.001, respectively], with prolonged RT and more errors in the incongruent condition compared to congruent condition.

#### The alerting by conflict interaction

The interaction between alerting and conflict was significant for RT (28 ± 51 ms, *t*(41) = 3.49; *p* < 0.001) but not for error rate [2.13 ± 9.05%, *t*(41) = 1.53; n. s.], indicating that the conflict effect on RT was greater in the no cue condition (206 ms) compared to double cue condition (179 ms).

#### The orienting by conflict interaction

The orienting by conflict interaction effects in RT (38 ± 44 ms) and error rate (5.04 ± 9.48%) were significant [*t*(41) = 5.58; *p* < 0.001 and *t*(41) = 3.45; *p* < 0.001, respectively] indicating that the conflict effect (RT = 141 ms, error rate = 6.32%) was significantly reduced by valid cues compared to double cues(RT = 179 ms, error rate = 11.36%).

#### The validity by conflict interaction

The validity by conflict interaction effects in RT (75 ± 42 ms) and error rate (9.41 ± 11.38%) were significant [*t*(41) = 11.67; *p* < 0.001 and *t*(41) = 5.36; *p* < 0.001, respectively], indicating that valid cues (RT = 141 ms, error rate = 6.32%) reduced the conflict effect compared to invalid cues (RT = 216 ms, error rate = 15.73%).

#### The correlation among the attentional effects

The correlation coefficients of the attentional effects in RT within the VS task are shown in the upper panel of **Table 4**. The overall RT was positively correlated with the conflict effect (*r* = 0.38; *p* < 0.01); the validity effect was positively correlated with the orienting effect (*r* = 0.67; *p* < 0.01), and with the orienting by conflict and validity by conflict interactions (*r* = 0.26; *p* < 0.05 and *r* = 0.32; *p* < 0.05, respectively). The alerting effect was positively correlated with the alerting by conflict interaction (*r* = 0.26; *p* < 0.05), and the orienting effect was positively correlated with the orienting by conflict interaction (*r* = 0.39; *p* < 0.01). Further, the validity by conflict interaction was positively correlated with the alerting by conflict interaction (*r* = 0.34; *p* < 0.01) and with the orienting by conflict interaction (*r* = 0.26; *p* < 0.05), while the orienting by conflict interaction was negatively correlated with the alerting by conflict interaction (*r* = -0.34; *p* < 0.05). Note that some of the effects are not independent because they share a common condition for the computation of attentional scores (e.g., alerting and alerting by conflict).

**Table 4:** Pearson correlation coefficients between the attentional effects (in RT) in the VS, AS, and AF tasks.

	Overall RT	Alerting	Orienting	Validity	Conflict	A × C	O × C
**VS**
Alerting	0.12							
Orienting	0.06	-0.22						
Validity	0.03	0.01	0.67^∗∗^					
Conflict	0.38^∗∗^	0.13	-0.11	0.17				
A × C	-0.12	0.26^∗^	-0.02	0.06	0.02			
O × C	0.17	-0.08	0.39^∗∗^	0.26^∗^	0.18	-0.34^∗^		
V × C	-0.14	0.10	0.21	0.32^∗^	0.07	0.34^∗^	0.26^∗^	
**AS**
Alerting	-0.32^∗^							
Orienting	0.12	-0.63^∗∗^						
Validity	0.02	-0.17	0.24					
Conflict	0.76^∗∗^	-0.15	0.02	0.10				
A × C	-0.30^∗^	0.61^∗∗^	-0.37^∗∗^	-0.14	-0.12			
O × C	0.26^∗^	-0.59^∗∗^	0.41^∗∗^	0.53^∗∗^	0.14	-0.91^∗∗^		
V × C	-0.07	-0.23	0.27^∗^	0.43^∗∗^	-0.07	-0.35^∗∗^	0.47^∗∗^	
**AF**	
Alerting	-0.11							
Orienting	-0.14	-0.29^∗∗^						
Validity	-0.18	0.22	0.36^∗∗^					
Conflict	0.53^∗∗^	-0.26^∗^	0.11	-0.21				
A × C	0.08	0.46^∗∗^	-0.22	0.10	0.13			
O × C	-0.13	-0.39^∗∗^	0.33^∗^	0.20	-0.19	-0.96^∗∗^		
V × C	-0.15	-0.21	0.02	0.07	0.14	0.03	-0.01	

A × C, alerting by conflict; O × C, orienting by conflict; V × C, validity by conflict; * *p* < 0.05; **p < 0.01.

### THE AUDITORY SPATIAL ATTENTION EFFECTS

**Figure 4** shows the network effects and the interactions in RT (center – top section) and error rate (center – bottom section) for the ANT-AS.

#### The alerting effect

The alerting effect was significant for both RT [61 ± 63 ms, *t*(41) = 6.33, *p* < 0.001] and error rate [2.53 ± 6.45%, *t*(41) = 2.5, *p* < 0.02], indicating that visual alerting improved both response speed and accuracy.

#### The orienting effect

The orienting effect was not significant for both RT [-1 ± 34 ms; *t*(41) = -0.98; n. s.] and error rate [0.07 ± 3.94%; *t*(41) = 0.11; n. s.], indicating that the AS orienting cue was ineffective.

#### The validity effect

The validity effect was not significant for both RT [-3 ± 47 ms; *t*(41) = -0.40; n. s.] and error rate [-0.13 ± 3.88%; *t*(41) = -0.22; n. s.], indicating no significant benefits following valid AS cues compared to invalid cues.

#### The conflict effect

The conflict effects in RT (179 ± 126 ms) and error rate (7.00 ± 8.95%) were significant [*t*(41) = 9.18, *p* < 0.001 and *t*(41) = 5.07, *p* < 0.001, respectively], indicating increased RT and error rate in the incongruent condition compared to the congruent condition.

#### The interactions

The alerting by conflict, orienting by conflict and validity by conflict interactions were not significant for both RT [*t*’s(41) = 1.08; -1.08; -1.19, n. s., respectively] and error rate [*t*’s(41) = 0.45; -0.11; 0.97, n. s., respectively].

#### The correlation among the attentional effects

The correlation coefficients of the attention effects in RT within the AS task are shown in the middle panel of **Table 4**. The overall RT was positively correlated with the conflict effect (*r* = 0.76; *p* < 0.01) and with the orienting by conflict interaction (*r* = 0.26; *p* < 0.05) while it was negatively correlated with the alerting effect (*r* = -0.32; *p* < 0.05) and with the alerting by conflict interaction (*r* = -0.30; *p* < 0.05). Further, the validity effect was positively correlated with the orienting by conflict (*r* = 0.53; *p* < 0.01) and validity by conflict (*r* = 0.43; *p* < 0.001) interactions. The alerting effect was positively correlated with the alerting by conflict interaction (*r* = 0.61; *p* < 0.001) and negatively correlated with the orienting effect (*r* = -0.63; *p* < 0.001) and with the orienting by conflict interaction (*r* = -0.59; *p* < 0.001). The orienting effect was positively correlated with the orienting by conflict and validity by conflict interactions (*r* = 0.41; *p* < 0.01 and *r* = 0.27; *p* < 0.05, respectively), while it was negatively correlated with the alerting by conflict interaction (*r* = -0.37; *p* < 0.01). Lastly, while the alerting by conflict interaction was negatively correlated with the validity by conflict (*r* = -0.35; *p* < 0.01) and orienting by conflict (*r* = -0.91; *p* < 0.01) interactions, these two interactions were positively correlated with each other (*r* = 0.47; *p* < 0.01).

### THE AUDITORY FREQUENCY ATTENTION EFFECTS

**Figure 4** shows the attention effects and the interactions calculated in RT (right – top section) and error rate (right – bottom section) for the ANT-AF.

#### The alerting effect

The alerting effect was significant for both RT [228 ± 126 ms, *t*(41) = 11.72, *p* < 0.001] and error rate [15.48 ± 6.04%, *t*(41) = 16.61, *p* < 0.001], indicating that auditory alerting cues improved response speed and accuracy.

#### The orienting effect

The orienting effect was significant for RT [21 ± 38 ms; *t*(41) = 3.66; *p* < 0.001] but not for error rate [0.45 ± 3.35%; *t*(41) = 0.87, n. s.], indicating that valid orienting cue effectively enhanced the response speed compared to the double cue condition.

#### The validity effect

The validity effects were significant in RT [16 ± 41 ms, *t*(41) = 2.54; *p* < 0.05] and error rate [1.44 ± 3.10%; *t*(41) = 3.01, *p* < 0.01], indicating shorter RT and greater accuracy in the valid cue condition compared to the invalid cue condition.

#### The conflict effect

The conflict effects in RT (100 ± 72 ms) and error rate (11.02 ± 5.64%) were significant [*t*(41) = 9.06, *p* < 0.001, and *t*(41) = 12.66, *p* < 0.001, respectively], indicating increased RT and error rate in the incongruent compared to congruent condition.

#### The alerting by conflict interaction

The interaction between alerting and conflict was significant for both RT [77 ± 136 ms, *t*(41) = 3.65; *p* < 0.001] and error rate [29.17 ± 14.90%, *t*(41) = 12.69; *p* < 0.001], indicating that the conflict effect (RT = 163 ms, error rate = 32.74%) was greater in the no cue condition compared to the double cue condition (RT = 86 ms, error rate = 3.57%). Alerting enhanced the conflict processing.

#### The orienting by conflict interaction

The orienting by conflict interaction was not significant for RT [7 ± 75 ms; (*t*(41) = 0.62; n. s.] and error rate [-0.03 ± 6.18%; *t*(41) = -0.04; n. s.].

#### The validity by conflict interaction

The validity by conflict interaction was not significant in RT [-6 ± 68 ms; *t*(41) = -0.61; n. s.] and error rate [0.56 ± 7.69%; *t*(41) = 0.48, n. s.].

#### The correlation among the attentional effects

The correlation coefficients of the attentional effects in RT within the ANT-AF are shown in the lower panel of **Table 4**. The overall RT was positively correlated with the conflict (*r* = 0.53; *p* < 0.01), as well as the alerting effect with the alerting by conflict interaction (*r* = 0.46; *p* < 0.01), the validity effect with the orienting effect (*r* = 0.36; *p* < 0.01), and the orienting with the orienting by conflict interaction (*r* = 0.33; *p* < 0.05). The alerting effect was negatively correlated with the orienting effect (*r* = -0.29; *p* < 0.05), with the conflict effect (*r* = -0.26; *p* < 0.05) and with the orienting by conflict interaction (*r* = -0.39; *p* < 0.01). The alerting by conflict and orienting by conflict interactions were negatively correlated (*r* = -0.96; *p* < 0.01).

### THE CORRELATION OF ATTENTIONAL EFFECTS ACROSS THE TASKS

**Table 5** shows Pearson’s correlation coefficients (top) and Spearman’s correlation coefficients (bottom) among the attentional effects in RT across the three tasks. Pearson’s correlation analyses showed that the overall RTs were correlated among the tasks (*r* = 0.53; *p* < 0.01 for VS and AS, *r* = 0.63; *p* < 0.01 for VS and AF, and *r* = 0.70; *p* < 0.01 for AS and AF). There were significant positive correlations between the conflict effects in the VS and AF ANTs (*r* = 0.32; *p* < 0.05) as well as between the two auditory ANTs (*r* = 0.67; *p* < 0.001), while the correlation for conflict effects between the VS and AS was not significant (*r* = 0.17; *p* = 0.15).

**Table 5:** Pearson’s correlation coefficients (top) and Spearman’s correlation coefficients (bottom) of the attentional effects (in RT) across the VS, AS, and AF tasks.

	VS and AS	VS and AF	AS and AF
**Pearson**
Overall RT	0.53^∗∗^	0.63^∗∗^	0.70^∗∗^
Alerting	-0.06	-0.2	0.14
Orienting	-0.04^a^	0.03	-0.22^a^
Validity	0.22^a^	-0.14	0.03^a^
Conflict	0.17	0.32^∗^	0.67^∗∗^
A × C	0.12	0.17	0.17
O × C	0.03	0.22	0.22
V × C	0.09	0.01	0.08
**Spearman**
Overall RT	0.53^∗∗^	0.58^∗∗^	0.67^∗∗^
Alerting	-0.08	-0.36	0.10
Orienting	-0.01	0.06	-0.21
Validity	0.33^∗^	-0.16	-0.02
Conflict	0.28^∗^	0.45^∗∗^	0.61^∗∗^
A × C	0.21	0.02	0.10
O × C	-0.11	0.22	0.16
V × C	0.01	-0.07	0.08

A × C, Alerting by Conflict; O × C, Orienting by Conflict; V × C, Validity by Conflict; *p < 0.05; **p < 0.01. ^a^The orienting and validity effects in the ANT-AS were not significant. Interpretations about correlations between these effects and other effects should be made with caution.

Spearman’s correlation analyses showed that the overall RTs were correlated among the tasks (*r* = 0.53; *p* < 0.001 for VS and AS, *r* = 0.58; *p* < 0.001 for VS and AF, and *r* = 0.67; *p* < 0.001 for AS and AF). There were significant positive correlations between the conflict effects in the VS and AS ANTs (*r* = 0.28; *p* < 0.05), between the conflict effects in the VS and AF ANTs (*r* = 0.45; *p* < 0.01), as well as between the conflict effects in the two auditory ANTs (*r* = 0.61; *p* < 0.01). The validity effects in the VS and AS were positively correlated (*r* = 0.33; *p* < 0.05), while the alerting effects in the VS and AF ANTs were negatively correlated (*r* = -0.36; *p* < 0.05).

### RELIABILITY OF THE ATTENTIONAL EFFECTS IN THE THREE TASKS

Split-half reliability coefficients of the attentional effects are reported in **Table 6**. For the ANT-VS, coefficients were 0.91 for the overall RT, 0.28 for the alerting effect, 0.19 for the orienting effect, 0.66 for the validity effect, 0.94 for the conflict effect, -0.36 for the alerting by conflict interaction, 0.30 for the orienting by conflict interaction, and -0.22 for the validity by conflict interaction. To account for the low reliability of some of our measures, we conducted split-half reliability analyses in two other datasets (Fan et al., 2009; Mackie et al., 2013). Results showed that the split-half reliability coefficients in Fan et al. (2009) and Mackie et al. (2013) were 0.42 and 0.45 for alerting, 0.41 and 0.49 for orienting, 0.59 and 0.51 for validity, 0.72 and 0.84 for flanker conflict, 0.25 and -0.27 for the alerting by conflict interaction, 0.13 and 0.01 for the orienting by conflict interaction, 0.56 and 0.48 for the validity by conflict interaction.

**Table 6:** Split-half reliability coefficients of the attentional effects in each task.

	Overall RT	Alerting	Orienting	Validity	Conflict	A × C	O × C	V × C
VS	0.91	0.28	0.19	0.66	0.94	-0.36	0.30	-0.22
AS	0.96	0.09	-0.55	0.07	0.95	-0.30	-0.11	-0.27
AF	0.96	0.81	0.30^1^	0.41	0.73	0.31	-0.21	0.41

VS, visuospatial; AS, auditory spatial; AF, auditory frequency; V × C, validity by conflict; A × C, alerting by conflict; O × C, orienting by conflict; ^*1*^computed based only on the long length target condition.

The coefficients for the ANT-AS were 0.96 for the overall RT, 0.09 for the alerting, -0.55 for the orienting, 0.07 for the validity, 0.95 for the conflict, -0.30 for the alerting by conflict interaction, -0.11 for the orienting by conflict interaction, and -0.27 for the validity by conflict interaction.

The coefficients for the ANT-AF were 0.96 for the overall RT, 0.81 for the alerting effect, -0.52 for the orienting effect, 0.41 for the validity effect, 0.73 for the conflict effect, 0.31 for the alerting by conflict interaction, -0.21 for the orienting by conflict interaction, and 0.41 for the validity by conflict interaction.

## DISCUSSION

In this study, we investigated the supramodal and modality-specific mechanisms of attentional functions by simultaneously testing the effects of the three attentional networks and their interactions, using a within-subjects design. Results suggested that executive control operates independent of sensory modality, while alerting and orienting functions may be implemented via modality-specific mechanisms. These results are consistent with neuroimaging studies showing the recruitment of the same frontoparietal network in response to both visual (Fan et al., 2005; Blasi et al., 2006) and auditory conflict tasks (Roberts and Hall, 2008; Donohue et al., 2012), as well as previous behavioral studies demonstrating modality-specific operation of the alerting (De Santis et al., 2007; Thiel and Fink, 2007) and orienting functions (Arnott et al., 2004; Kong et al., 2012; Larson and Lee, 2014).

As previously mentioned, the attentional functions rely upon both the *site* of sensory encoding for a given modality (i.e., primary cortex) and the *source* of attentional control. The sources may be supramodal while the sites must be modality-specific. Consequently, observed correlations across modalities may reflect shared source and/or site (e.g., for the executive control network). Conversely, a lack of correlation across modalities may reflect a similar source but different attentional sites (e.g., for alerting and orienting). We were unable to separate the effects of site and source in this study and the discussion presented below takes this limitation into account.

### MODALITY-SPECIFIC MECHANISMS OF ALERTING AND ORIENTING

The finding that alerting and orienting appear to operate via modality-specific mechanisms may be explained by how information is processed at lower levels within these modalities (i.e., the site). In contrast to the encoding of space in vision, where spatial information is extracted directly from the layout of the retina, the auditory cortex is organized tonotopically and spatial information is computed indirectly from signal differences between the two cochleae (Bilecen et al., 1998; Kong et al., 2012). Thus alerting, which occurs in response to a signaling cue, may depend upon the processes in which the primary cortex encodes sensory information in order to relay it to higher-level areas responsible for salience detection and signal the phasic alerting response (Posner, 1978). Modality-specific alerting mechanisms may decrease reactivity in other modalities in order to optimize responsiveness toward objects of interest in the real world. Furthermore, while an auditory warning requires no active attention in order to produce its alerting effect, visual cues produce alerting only if attention is turned toward the stimulus (Posner, 1978). Differences between visual and auditory attentional functions may also exist for orienting, wherein the function is closely tied to the specific modality in which stimuli are presented (Woodruff et al., 1996; O’Leary et al., 1997; Johnson and Zatorre, 2005; Ahveninen et al., 2006; Larson and Lee, 2014).

Modality-specific attention mechanisms can be further explained by the possible adaptive benefits when facing complex environments. Once attentional resources are directed to one sensory modality, e.g., toward an alerting sound suddenly presented, another alerting stimulus presented close in time in a different modality would theoretically compete for the same attentional resources. There is some evidence that this is not the case, as the simultaneous presentation of both visual and auditory warning cues in a previous study provided no additional benefits or costs, compared to when each warning cue was displayed alone (Fernandez-Duque and Posner, 1997). The existence of modality-specific alerting and orienting mechanisms is in line with evidence showing that attentional resources can be divided across competing tasks that are in different modalities, such as shadowing an auditory stream while also attending to the presentation of visual words (Allport et al., 1972). Similarly, orienting mechanisms toward stimuli in different modalities should not interfere with each other, as this would result in inefficient interactions in real-world situations.

### EXECUTIVE CONTROL OF ATTENTION IS SUPRAMODAL

Executive control of attention involves the engagement of complex mental operations during the detection and resolution of conflicts between competing mental processes (Posner and Petersen, 1990; Fan et al., 2003; Mackie et al., 2013). Achieving dynamic control of behavior in complex environments requires a mechanism that coordinates the processing of information across different modalities. Positive correlations of conflict effects among the visual and auditory tasks indicate that shared executive control resources are employed across the two modalities.

Executive control is typically thought of as a higher-level function not tied to any particular modality (Mackie et al., 2013; Fan, 2014). It is integrative across modalities, allowing for flexible responses to complex situations. Supramodal executive control, along with modality-specific alerting and orienting, supports the idea of a hierarchy of functions among the three attentional networks, with executive control being at higher-order relative to the alerting and orienting functions (Wang and Fan, 2007). There is a possible adaptive benefit of modality-independent executive control of attention. Having controllers within each modality would reduce efficiency because additional cross modality coordination at a higher level would still be required. The observed correlated effects between the visual and auditory tasks indicate that executive control recruits shared sources across modalities. Further, we observed that the conflict effect was highly correlated with overall RT across tasks, indicating a possible common latent source.

### CONSIDERATIONS ABOUT THE RELIABILITY OF THE TASKS

For the ANT-VS, the orienting effect appeared to be the least reliable, the conflict effect was the most reliable, and the alerting and validity effects had intermediate reliability. To further understand the low reliability of some of our attentional effects, split-half analyses were also conducted on two other ANT-R datasets (Fan et al., 2009; Mackie et al., 2013). Results showed that the split-half reliability of the alerting and orienting effects in the ANT-R was greater than that reported with the ANT-VS, which may be related to a change in the interval between cue and target used in this study.

The reliability of the alerting and orienting for the auditory tasks was low, especially for the ANT-AS. This may in part be due to the use of orthogonal subtractions of performance between specific conditions, which is associated with a doubled variance and therefore a reduction in the reliability of these network measures. The low reliability of the alerting and orienting functions may be inherent to the existence of modality-specific mechanisms, for example, the greater variability of these effects during the auditory tasks as suggested by the variation of the orienting effect in the ANT-AF, make them more difficult to accurately quantify. An alternative explanation for the low correlation of the alerting and orienting effects across modalities may be related to the low reliability (see the related section below). In this sense, further investigation is warranted to examine whether or not these attentional functions are supramodal. The existence of distinct “what” and “where” cortical pathways (Ahveninen et al., 2006), areas of activation (e.g., Thiel and Fink, 2007; Kong et al., 2012; Larson and Lee, 2014), a previous behavioral study (Lord et al., 1968; Roberts et al., 2006), in addition to our results, provide evidence which points to the influence of modality-specific processes in alerting and orienting functions. Regarding executive control, the effects were reliable across tasks, which strengthens our conclusion related to the existence of a supramodal executive control of attention.

The purpose for designing the ANT-AS was to test whether the alerting and orienting effects in the AS modality are correlated with the VS effects measured by the ANT-VS. However, this new ANT-AS version did not provide reliable measures of both alerting and orienting functions. Therefore, future studies aiming to test the efficiency and interaction of the attentional networks in the AS modality should improve upon this version of the ANT by using spatially distributed speakers instead of headphones to present spatial cues in a more ecologically valid manner. It is possible that such a manipulation would still not be effective, due to the relatively lower precision of and greater effort required for auditory localization in comparison to visual localization. Reliable attentional effects were found for the ANT-AF. There was a negative split-half reliability coefficient of the orienting effect in the ANT-AS. A negative reliability coefficient can occur when the two halves are not parallel (i.e., each subject has a different true score on both measures or the error variances of the two measures are not equal). It is unlikely that the unstable orienting effect found for the AS task depicts a situation in which the auditory orienting of attention in humans is unreliable, which would be the case if each subject had different true orienting scores in each half. Therefore, a negative reliability coefficient could be obtained for this task due to the sum of the variances being greater than the variance of the true orienting effect. Reducing error variance in measurements would improve the task and produce a more reliable measure of AS orienting. Within the current design, the ANT-AF is better than the ANT-AS in terms of reliability to measure the efficiency of the attentional functions in the auditory modality.

### CONCLUSION

In summary, we found significant correlations for executive control effects in the visual and auditory modalities. However, we did not find significant correlations for the alerting and orienting effects. Our findings point toward the existence of supramodal mechanisms for the executive control of attention, while alerting and orienting functions may rely more upon modality-specific processes.

## Conflict of Interest Statement

The authors declare that the research was conducted in the absence of any commercial or financial relationships that could be construed as a potential conflict of interest.
